# Systemic and local evidence for complement involvement in chronic spontaneous urticaria

**DOI:** 10.1002/clt2.12011

**Published:** 2021-07-03

**Authors:** Mehran Alizadeh Aghdam, Mignon van den Elzen, Harmieke van Os‐Medendorp, Marijke R. van Dijk, Edward F. Knol, André C. Knulst, Heike Röckmann, Henny G. Otten

**Affiliations:** ^1^ Department of Dermatology/Allergology UMC Utrecht Utrecht The Netherlands; ^2^ Department of Pathology UMC Utrecht Utrecht The Netherlands; ^3^ Department of Immunology UMC Utrecht Utrecht The Netherlands

**Keywords:** blood, complement, omalizumab, skin, urticaria

## Abstract

**Background:**

The pathogenesis of chronic spontaneous urticaria (CSU), including the mechanism of action of omalizumab, remain unclear. We hypothesized complement system involvement given the often fast clinical response induced by treatment, including omalizumab. Therefore, we assessed the role of various complement factors surrounding omalizumab treatment.

**Methods:**

Thirty CSU patients (median age 42 [range 21–70]; 73 % female) with a median once daily Urticaria Activity Score over 7 days (UAS7) score at baseline of 31.5 points were enrolled. Treatment consisted of six administrations of 300 mg omalizumab every 4 weeks succeeded by a follow‐up period of 12 weeks. Four punch skin biopsies were taken per patient; at baseline from lesional skin, at baseline from nonlesional skin, and after 1 and 7 days from formerly lesional skin. Complement activity, including C1q, C3, C3bc/C3, C4, C4bc/C4, C5a, and Membrane Attack Complex in peripheral blood were analyzed and complement activation in the skin was determined by the analysis of C4d deposition. Results were related to the clinical response to omalizumab.

**Results:**

Fifteen patients showed a UAS7 score of 6 or lower (median 0) at Week 24, 15 patients did not (median 16). Lesional skin biopsies at baseline revealed complement deposition (C4d) in blood vessels in the papillary dermis of 53% (16/30) of the patients, which suggests involvement of immune complexes in the pathogenesis of urticaria. Moreover, indication of increased complement activation in CSU was substantiated by increased C5a levels in peripheral blood compared to healthy controls (*p* = 0.010). The clinical effect of omalizumab could not be linked to the variation of complement components.

**Conclusions:**

Both C4d deposition in lesional skin and elevated C5a levels in peripheral blood indicate the involvement of complement activation in the pathogenesis of CSU. No correlation was found between omalizumab and activation of complement indicative of independent processes in the immunopathogenesis of CSU.

## 1 INTRODUCTION

Chronic spontaneous urticaria manifests as a burdensome skin disease with sudden onset, sometimes severe itching and wheals, that lasts for at least 6 weeks. Prevalence is estimated to be up to 1% at any time, with disease duration ranging from 1 to 5 years or even longer in more severe cases.^1^ Omalizumab has a reported clinical response of over 50% within the first 2 days of treatment in CSU patients.^2^ Depletion of free IgE by omalizumab leads to downregulation of the FcεRI on mast cells^3^ and basophils^4^ in patients with allergic disease.^5^ However, this downregulation alone cannot explain the fast clinical response to omalizumab. Hence, alternative mechanisms must be contributing to the rapid clinical efficacy of omalizumab.

Sera from patients with urticaria can induce degranulation of basophils, a process in which the presence of intact complement and patient IgG containing specific antibodies against IgE or the high affinity IgE receptor is essential.^6^ By binding to IgE or FcεRI on mast cells, complement via the classical pathway can be activated and lead to the generation of C5a and C5b‐9.^7^ C5a can subsequently bind to the complement C5a receptor on mast cells and cause degranulation. Cutaneous mast cells express the complement C5a receptor whereas mucosal mast cells do not. Additionally, the complement system is known for its rapid response upon activation. This may explain how IgG anti‐FcεRI autoantibodies in combination with complement in patients with CSU can cause fast clinical symptoms which are limited to the skin and not mucosal tissue.^8^

It has been reported that C1q, C2, C3, C4, and C5 levels in peripheral blood are within normal limits in chronic urticaria but no data have been published regarding complement degradation/activation products in peripheral blood .^9^, ^10^, ^11^ The effect of omalizumab treatment on peripheral blood complement levels in CSU patients has also never been studied.

Furthermore, it is unknown whether complement activation occurs in the skin of patients with CSU. Complement activation in tissue can be evaluated by determination of C4d deposition: a well‐studied marker and a characteristic feature of complement activation, which is, for instance, also included in the BANFF criteria for humoral rejection after kidney transplantation.^12^ In this study, we investigated the role of the complement system and the effects of omalizumab treatment in CSU patients using the C4d marker in skin and peripheral blood samples. Additionally, we hypothesize that the efficacy of omalizumab in CSU may in part be accompanied by reduction of complement mediated inflammation.

## 2 METHODS

### 2.1 Design and population

This monocenter exploratory prospective cohort study was performed in the University Medical Center Utrecht, The Netherlands, from 2015 to 2017. Inclusion criteria were adult CSU patients with a significant disease activity defined as a once daily Urticaria Activity Score over 7 days (UAS7) ≥ 16 and a UAS7 ≥ 4 on the day of the first omalizumab administration despite treatment with antihistamines up to four times the daily dose. Exclusion criteria were based on one of the pivotal randomized controlled trials^13^ and included a clearly defined underlying etiology for chronic urticaria (e.g., chronic inducible urticaria), a history of malignancy, known hypersensitivity to omalizumab, and pregnancy. Routine administration of immunosuppressants including prednisolone and ciclosporin^14^ were discontinued with washout periods of 3 months prior to treatment with omalizumab. If prednisolone was used as rescue medication, a washout period of 2 weeks was maintained before the start of the study. After a screening period of up to 2 weeks, eligible patients started a 6‐month treatment period, followed by a follow‐up period of 3 months. The latter could be shortened upon patient‐request if the UAS7 was projected to reach a score of 16 or higher. All patients provided written informed consent, and the study was approved by the local ethics committee (protocol number 15‐167).

### 2.2 Omalizumab and concomitant medication

All patients received six doses of 300 mg omalizumab every 4 weeks with follow‐up starting at Week 25, 4 weeks after the last dose. Leukotriene receptor antagonists or H2 blockers for indications other than CSU were permitted to be continued during the study. Patients were allowed to use H1‐antihistamines up to a maximum of four doses per day as rescue medication in addition to their concomitant medication, as well as prednisolone up to 30 mg. Due to worsening of the disease, 11 patients, of which 6 (55%) were presented as responders, restarted omalizumab treatment during follow‐up. Data of subjects who restarted omalizumab during the follow‐up period were removed from data analysis from that consecutive time‐point. In absolute numbers, the number of patients who restarted omalizumab were: one in week 25, two in week 26, three in week 28, four in week 29, nine in week 30, and 11 in week 32.

### 2.3 Assessments in blood samples

Blood samples were collected at the following time‐points: at baseline, after 1, 2, 6, and 24 h, after 1 and 2 weeks, and 4 weeks after the first administration of omalizumab. Subsequently, blood was collected prior to each subsequent dose. Lastly, a venipuncture was performed at the last follow‐up visit. For measurement of complement activation, ethylenediaminetetraacetic acid (EDTA) plasma, serum, and gel separated serum were used. EDTA blood and gel separated serum were put on ice immediately after venipunctures. All serum samples were allowed to coagulate for 60 min. Serum and plasma were obtained by centrifugation and stored at −80°C.

### 2.4 Measurements complement

Complement levels of C3 and C4 were determined in serum by an immunonephelometric method on a SPA+ turbidimeter, C5b‐9 membrane attack complex formation via the classical complement activation route was measured in gel separated serum using a commercially available enzyme‐linked immunosorbent assay (EuroDiagnostica) according to the manufacturer's recommendations, and C5a was determined in EDTA plasma via Luminex xMAP technology (Luminex Corporation). Additionally, C1q in serum, and C3bc and C4bc in EDTA plasma were determined as previously described.^15^

### 2.5 Skin biopsies

**FIGURE 1:**
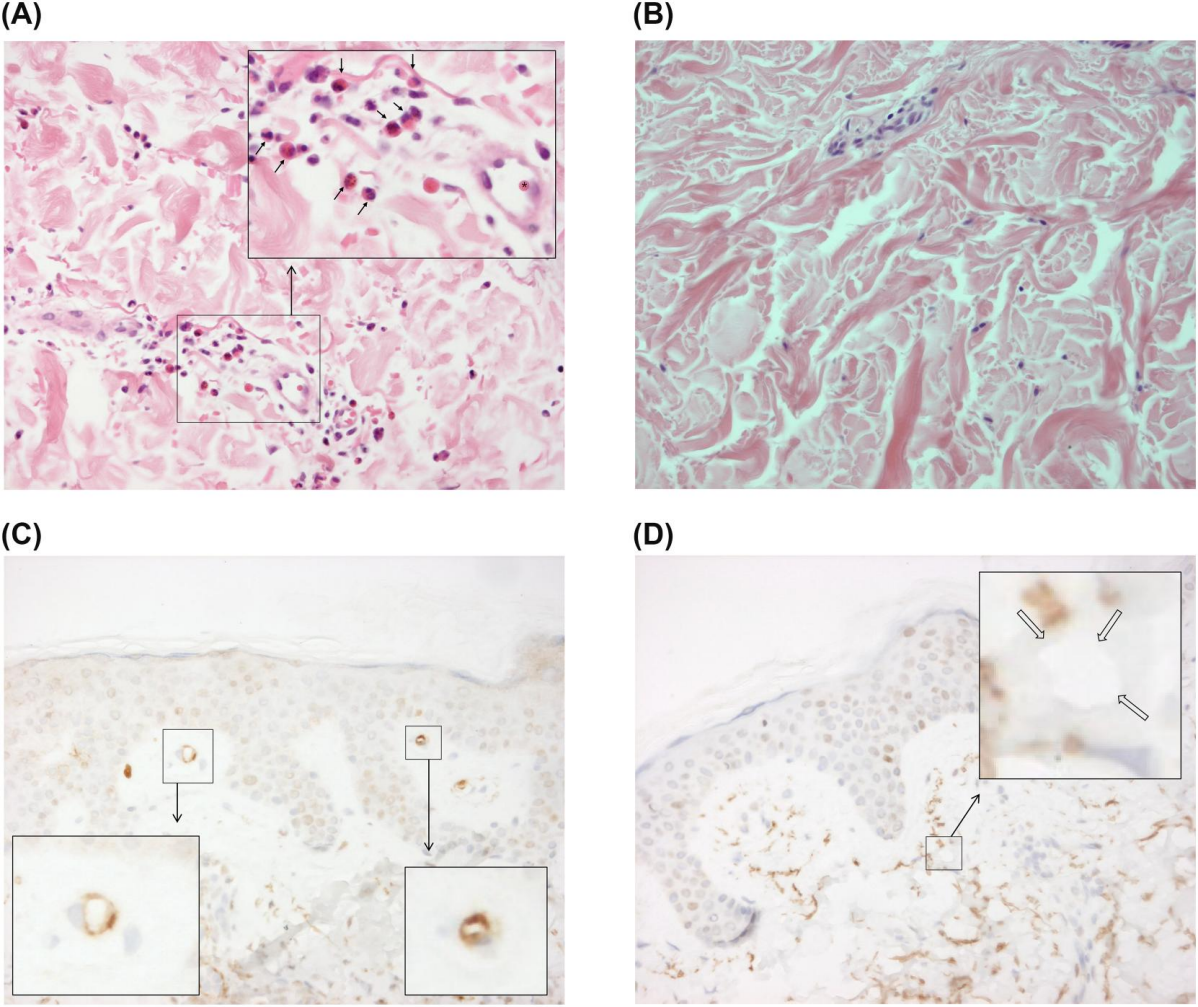
Inflammation and C4d deposition in skin. (A) HE staining with evident edema, perivascular infiltration, limited interstitial infiltration, and many eosinophils. Arrows indicate eosinophils; *: erythrocyte within blood vessel. (B) HE staining with very limited edema, no infiltration, and absence of granulocytes. (C) C4d staining with evident c4d deposition fully surrounding vessel walls. (D) C4d staining, open arrows indicate blood vessels with absence of C4d

A total of four 3 mm punch skin biopsies were taken per patient: (1) at baseline from lesional skin; (2) at baseline from nonlesional skin, and after 1 (3) and 7 days (4) from formerly lesional skin. Skin sections were formalin‐fixed, paraffin‐embedded, and stained by immunohistochemistry with specific antibodies allowing characterization of inflammation (HE‐staining), CD3 (DAKO), CD4 (Cellmarque), CD8 (DAKO), CD20 (Roche), CD68 (Leica), CD138 (Serotec), and 2D7‐antibody (Hycult). All characteristics were visually examined and judged on a 0–3 semiquantitative scale^16^, ^17^ as “not elevated” (0) or a mild, moderate, or severe increase (1–3), on original magnification ×400. Complement activation in the skin was evaluated by determination of C4d deposition (polyclonal rabbit anti‐C4d staining; ALPCO), and was graded from 0 (negative) to 3 (bright signal or fully surrounding blood vessel walls) (Figure 1). As previously described, the original magnification was ×400.^17^

### 2.6 Clinical assessments and patient‐reported outcomes

Disease activity was measured throughout the study by using the UAS7.^18^ Missing daily scores of the weekly disease activity scores after treatment was started were complemented by Last Observation Carried Forward method up to a maximum of 3 days. Missing follow‐up scores were supplemented with data from each patient's clinical record following clinical visits if available. Weekly scores that could not be complemented with earlier mentioned methods were marked as missing and were not included in patient reported outcome results. Disease control was measured at baseline, 4 weeks after each administration, and at the last follow‐up visit by using the urticaria control test.^19^

Treatment response has been defined as a UAS ≤ 6 at Week 24 of treatment.

### 2.7 Statistical analysis

Changes in inflammatory parameters in the skin were related to changes in levels of circulating complement components, by using Spearman Rank correlation. Inflammatory characteristic after treatment were compared to baseline, and/or to the previous measurement, using Wilcoxon matched pairs signed rank tests, or paired samples *T* test as appropriate. C3bc and C4bc activation ratios were determined by dividing the level of circulating C3bc or C4bc by the amount of C3 or C4, respectively, and multiplying the quotient by 100 to determine the percentage, as previously described.^15^ Study population baseline scores for C5a were compared with normal values of C5a (based on results in a pool of 43 healthy volunteers) using the Mann–Whitney *U* test. For all statistical tests, a *p*‐value of 0.05 or lower was considered significant.

Descriptive analyses were carried out for all clinical efficacy outcomes. For each protein, Spearman's correlations were calculated between the difference in complement level from baseline after 1 h (C5a: after 2 h) and the difference in UAS7 score from baseline after 1 week. Additionally, the change from baseline in peripheral blood complement components was related to the change from baseline in UAS7, also by using the Spearman Rank correlation or Pearson correlation if appropriate. Statistical analysis was performed using IBM SPSS Statistics version 21 or GraphPad Prism version 7.02, graphs were prepared using Microsoft Visio 2010 or GraphPad Prism version 7.02.

## 3 RESULTS

### 3.1 Population

In this study, 30 patients (median age 42 [range 21–70]; 73 % female) with a median UAS7 score at baseline of 31.5 points were enrolled. Twelve of the thirty patients (40%) reported concomitant CindU complaints while 24 of the 30 patients (80%) reported an history of angioedema attacks. The median disease duration was 2.7 years (range 0.6–29). Detailed clinical characteristics can be found elsewhere^4^ Overall, patient characteristics corresponded with the CSU population in our clinic and current literature.^20^ Fifteen patients (50%) showed a UAS7 score of 6 or lower (median 0) at Week 24 (4 weeks after the last omalizumab administration) and were defined as responders. When analyzing response by the use of the minimal important difference (MID) of 10 UAS7 points, 23 patients (76,6%) were responder at Week 24, which included nine complete responders (UAS7 = 0) , which is fairly similar to daily practice data. Four of the 30 patients (one responder, three nonresponders) reported prednisolone use at some time‐points during the study period.

### 3.2 Inflammation and complement activation in lesional and nonlesional skin

Quantification of histological alteration found in skin biopsies of patients compared to healthy controls is presented in Table 1. Histological analysis demonstrated no significant differences between lesional and nonlesional biopsies at baseline or follow‐up with regard to edema and cellular infiltration. Higher amounts of C4d deposition were significantly more frequently found in lesional skin compared to nonlesional skin (*p* = 0.033) (Table 1). In the total 60 baseline skin biopsies, there was a significant correlation between C4d deposition and eosinophils scores (Spearman's ρ 0.358; *p* = 0.005).

**TABLE 1:** Inflammation and complement deposition in skin

Frequency of dermal changes	Lesional baseline				Nonlesional baseline				Lesional Day 1				Lesional Day 7			
	0	1	2	3	0	1	2	3	0	1	2	3	0	1	2	3
Edema																
Superficial	14	13	2	0	22	7	1	0	23	7	0	0	25	5	0	0
Deep dermis	16	9	4	0	20	9	1	0	20	8	2	0	23	7	0	0
Perivascular infiltration																
Superficial	7	19	3	1	9	21	0	0	13	15	2	0	13	15	2	0
Deep dermis	25	3	2	0	29	1	0	0	29	1	0	0	28	1	1	0
Interstitial infiltration																
Superficial	20	7	3	0	25	5	0	0	28	2	0	0	27	3	0	0
Deep dermis	19	6	3	2	27	3	0	0	26	2	1	1	27	3	0	0
T‐cells																
CD3	4	22	3	0	5	23	2	0	5	21	4	0	7	19	4	0
CD4	3	19	8	0	3	16	11	0	4	16	9	0	5	16	9	0
CD8	16	14	0	0	15	15	0	0	15	14	0	0	17	13	0	0
B‐cells																
CD20	27	3	0	0	29	1	0	0	30	0	0	0	29	1	0	0
Plasma cells	27	1	0	0	28	1	0	0	29	1	0	0	28	1	0	0
Granulocytes																
Neutrophils	14	7	7	1	26	3	1	0	24	4	1	1	27	3	0	0
Eosinophils	17	5	5	3	26	2	0	2	24	5	0	1	30	0	0	0
Basophils	24	3	2	1	27	1	1	1	27	2	1	0	29	1	0	0
Mast cells	23	6	1	0	26	4	0	0	22	7	1	0	24	6	0	0
Histiocytes	5	18	7	0	11	19	0	0	11	15	3	1	16	12	2	0
C4d deposition	13	5	4	7	17	8	4	1	19	8	1	2	19	7	3	1

*Note*: Score 0, not elevated; 1–3, mild, moderate, or severe increase compared to healthy skin. It was not possible to make a reliable assessment of all items in seven of 120 biopsies, therefore not all characteristics add up to 30 patients.

### 3.3 Complement activation in peripheral blood

**FIGURE 2:**
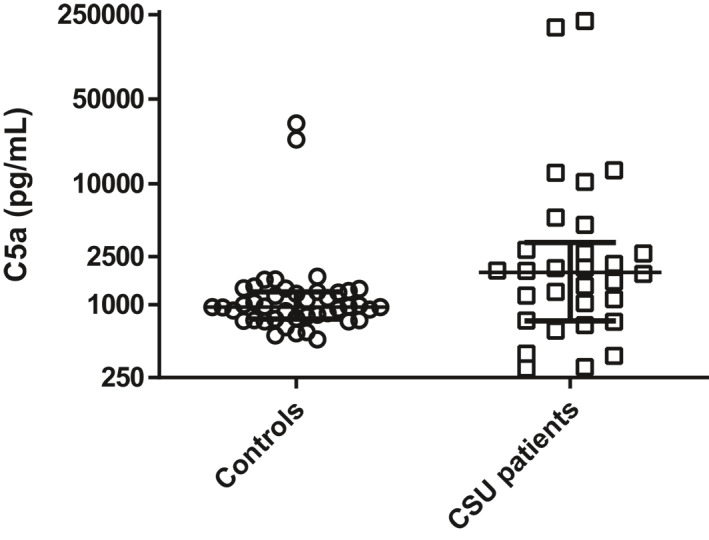
C5a levels at baseline of healthy controls compared to CSU patients. Data are shown as median C5a levels (IQR)

Table 2 shows that in a large portion of patients, peripheral blood levels of all complement components investigated were within normal ranges throughout the study. Most investigated complement component levels in peripheral blood were within normal ranges at both baseline and throughout the study. However, as shown in Figure 2, C5a levels at baseline (median 1847,6 pg/ml) were increased compared to healthy controls (median 959.2 pg/ml; *p* = 0.010). No disproportion was seen for aberrant values since they were either too high or too low in an equal proportion of patients. For example, C1q levels at baseline were reduced in three patients (10%) and elevated in 8 (27%), and throughout the study 32 (8%) C1q measurements were reduced and 61 were elevated (16%). Additionally, no correlations were found between complement component levels in peripheral blood and C4d deposition in skin.

**TABLE 2:** Peripheral blood complement component levels

Protein	Normal value	Baseline measurements Reduced *n* (%) Elevated *n* (%)		Total measurements Reduced Elevated values (%) values (%)	
C1q	81–128 IU/ml	3/30 (10)	8/30 (27)	32/352 (9)	61/352 (17)
C3	0.9–1.8 g/L	1/30 (3)	0	21/352 (6)	0
C4	0.1–0.47 g/L	1/30 (3)	0	12/352 (3)	0
C5a	<13605 pg/ml	NA	2/30 (7)	NA	9/250 (4)
MAC	69–129%	5/30 (17)	1/30 (3)	21/238 (9)	5/238 (2)

*Note*: Aberrant complement measurements for the number of patients (*n*) are shown at baseline and for all cumulative measurements for the following timepoints. Number of missing values C1q:8, C3:8, C4:8, C5a:8, MAC:5. ‘Reduced” and “Elevated” indicate values below lower limit of normal, or above upper limit of normal.

Abbreviation: MAC, C5b‐9 membrane attack complex formation.

Peripheral blood complement component measurements within two hours after omalizumab injection were disregarded, since a short‐term decrease of less than 10% in complement components are a known phenomenon after biological treatment.^21^ No differences in complement levels were found after 2 h for all other time points compared to baseline. When comparing nonresponders and responders, no statistical difference could be found in the complement component levels and their degradation products. No statistical significant correlations between difference in disease activity after 1 week and difference in peripheral blood complement levels 1 h after the first administration were found (see Table A1). Additionally, no correlations were found between UAS7 after 1 week and both complement levels and difference in complement levels after 1 h.

## 4 DISCUSSION

In this study, we investigated the role of the complement system in skin and peripheral blood in 30 patients with chronic spontaneous urticaria treated with omalizumab. Treatment with omalizumab resulted in a strong improvement in median disease activity, which was in line with previous studies.^20^

This is the first study to demonstrate activation of the complement system in the both peripheral blood and skin of CSU patients. At baseline, we found complement C4d deposition in the lesional skin in 53% of the patients, and significantly higher peripheral blood C5a levels in CSU patients compared to healthy individuals, indicating complement activation in a significant percentage of CSU patients. Within the first hour after first omalizumab administration, different complement components in the peripheral blood decreased irrespective of disease activity or treatment response, indicating that omalizumab administration leads to consumption of complement components. No relation was found between complement components investigated and disease activity (UAS7) scores prior to or after treatment with omalizumab, indicating that the clinical responses induced by omalizumab are not related to short‐ or long‐term changes in circulating levels of complement components and their degradation products.

To the best of our knowledge, it is a novel finding that C4d is present in small blood vessel walls within the papillary dermis of a majority of CSU patients. This finding suggests that IgG or IgM autoantibodies in the skin are able to cause complement activation and supports the current hypothesis that IgG autoantibodies are involved in the pathogenesis of CSU.^3^, ^22^ Complement‐fixing autoantibodies and complement deposition in the skin are also frequently found in systemic lupus erythematosus (SLE), which may point to common pathomechanisms in CSU and SLE.^23^ In SLE, C4d was found not only in blood vessel walls (80% of patients) but also along the dermoepidermal junction (100% of patients). In the subacute cutaneous lupus erythematosus, deposits of C4d were detected within epidermal keratinocytes, and in pemphigus cases, intercellular C4d was found which roughly corresponded to the location of autoantibodies.^24^ The location of C4d deposition—in the superficial dermis—is where infiltration was seen most in patients with urticaria. It is not surprising that C4d deposits were, in lower amount, also present in nonlesional skin, as the presence of C4d deposition in nonlesional skin may be indicative of previous whealing—the nonlesional skin might in fact be postlesional, since urticarial lesions tend to come and go—or it may point toward a widespread rather than local activation of the complement system. At present, it is unknown whether C4d deposition in urticaria is limited to the skin.

Furthermore, the correlation trend between C4d deposition and neutrophils scores and perivascular infiltration scores in the superficial dermis suggests granulocyte infiltration might be accompanied by local complement activation.

The role of complement in CSU is further supported by the fact that baseline C5a levels in peripheral blood were elevated in CSU patients compared to healthy individuals. Since it is known that complement activation and in particular C5a can be important for basophil activation in urticaria,^25^, ^26^ these results support the role of complement in pathogenesis of CSU. We observed no correlation between the presence of C4d and C5a levels. This may be explained by the fact that C4d binds covalently and remains stable in structures surrounding endothelium, thus escaping early removal from the target organ whereas C5a is cleared rapidly. In patients recovering from acute humoral rejection after kidney transplantation, C4d deposition was cleared after 21–41 days,^27^ in part due to plasmapheresis, which is continued until circulating IgG antidonor HLA‐antibody levels are sufficiently reduced. In our study, we found a much faster decrease of C4d deposition in the skin. This situation is not comparable to acute humoral rejection as remaining IgG HLA‐antibodies present in the kidney tissue will influence the rate of C4d clearance, whereas omalizumab reduces IgE and not IgG levels.^28^ Since complement is mainly activated by IgG,^29^ omalizumab treatment might not have an influence on the complement system as this drug exclusively binds IgE which cannot fix complement. This suggest that, in at least a proportion of patients with CSU, the relevance of complement in the pathogenesis is minimal.

Upon omalizumab administration complement activation was found within an hour and of C5a within 2 h, after which all levels normalized within 6 h. Immediate response of the complement system has been shown before in rituximab, omalizumab, and OKT3 treatment where complement consumption could be observed already within 5 min after completion of rituximab infusion^21^ and an increase in C3bc or C4bc was observed 30 min after onset of infusion.^30^ Hence, this immediate complement activation is not specific to omalizumab. Complement activation at baseline may be caused by autoantibodies known to be commonly present in CSU patients although it must be noted that recent studies show contemporary IgE and IgG responses to the same autoantigens.^3^, ^22^, ^31^, ^32^, ^33^ Little is known about these autoantibodies. The incidence of thyroid autoantibodies in patients with chronic urticaria is reported to range from 6.5% to 57% However, whether these antibodies predispose to autoimmune thyroiditis and hypothyroidism is not clear. Additionally, specific antinuclear antibodies have been studied, but a low frequency of positivity was reported (2.5% of women and 0.9% of men), and again, the relation to clinical symptoms remains unknown.^34^ Furthermore, as C5a normalization was not persistent throughout the study, we conclude that the early clinical responses observed after administration are not due to restoration of complement‐mediated pathophysiology in CSU. The question remains how this temporary complement activation upon anti‐IgE therapy can be explained. Baseline demographic characteristics were fairly similar to previous studies, and therefore we expect that our results are generalizable to the general CSU population in need of third‐line treatment. Previously, Kolkhir et al.^35^ reviewed the hypothesis of Type I and Type IIb autoimmunity in the pathogenesis of CSU. The authors discuss that an IgG‐anti‐FcɛRI/IgE‐mediated activation of mast cells and basophils might be depended of complement C5a through its C5aR receptor. Furthermore, in SLE systemic formation of complexes of C1q and C3 with IgG is seen as deposition of immune complexes along the dermal‐epidermal border, a phenomenon which might be reflected by C4d deposition in CSU patients.^23^

The relatively small patient numbers and the absence of a multiple testing correction for the different complement components is a limitation of this study. Therefore, additional research in larger study populations are needed. In conclusion, both C4d deposition in lesional skin and elevated C5a levels in peripheral blood indicate the involvement of complement activation in the pathogenesis of CSU. No correlation was found between (response to) omalizumab and activation of complement, indicative of independent processes in the immunopathogenesis of CSU.

## CONFLICT OF INTERESTS

Mignon van den Elzen received reimbursements to attend symposia and speaker's fees from Novartis Pharma B.V. to the institution. André C. Knulst received funds for research and healthcare innovation from Novartis Pharma B.V. to the institution and was involved in the advisory board of Novartis Pharma B.V. Heike Röckmann received speaker's fees, and funds for research from Novartis Pharma B.V. to the institution. Henny G. Otten received funds for this research from Novartis Pharma B.V. to the institution. The other authors declare that there are no conflict of interests.

## ETHICS STATEMENT

All patients provided written informed consent, including consent for publication. The study was approved by the local ethics committee (protocol number 15‐167).

## Supporting information

Supplementary Material
