# Supplementary material for: Systemic and local evidence for complement involvement in chronic spontaneous urticaria
Source: Clin Transl Allergy. 2021 Jul 3;11(5):e12011. doi: 10.1002/clt2.12011 (PMC8254579; doi:10.1002/clt2.12011)
Supplement: Supplementary file 1 — Supplementary Material [file CLT2-11-e12011-s001.docx]

| Protein | Correlation with UAS7  ρ (p-value) |
| --- | --- |
| C1q | 0.059 (0.765) |
| C3 | -0.147 (0.454) |
| C3bc/C3 | 0.345 (0.072) |
| C4 | -0.126 (0.524) |
| C4bc/C4 | 0.153 (0.438) |
| C5a | -0.038 (0.848) |
| MAC | 0.159 (0.428) |

**Table E1: correlations between difference in disease activity after 1 week and difference in peripheral blood complement levels 1 hour after the first administration**

For each protein Spearman’s correlations were calculated between the difference in complement level from baseline after 1 hour (C5a: after 2 hours) and the difference in UAS7 score from baseline after 1 week.

Total number of measurements: 390 for C1q, C3, and C4, and 276 for C3bc, C4bc, C5a, and MAC.
